# Dilatant Failure States for Drained Triaxial Compression of Some Geomaterials

**DOI:** 10.3390/ma18225181

**Published:** 2025-11-14

**Authors:** Zenon Szypcio, Katarzyna Dołżyk-Szypcio, Katarzyna Gabryś, Wojciech Sas

**Affiliations:** 1 Department of Geotechnics, Roads and Geodesy, Faculty of Civil Engineering and Environmental Sciences, Bialystok University of Technology, 45A Wiejska Street, 15-351 Bialystok, Poland; k.dolzyk@pb.edu.pl; 2 Department of Geotechnics, Institute of Civil Engineering, Warsaw University of Life Sciences—SGGW, 159 Nowoursynowska Street, 02-776 Warsaw, Poland; wojciech_sas@sggw.edu.pl

**Keywords:** geomaterials, triaxial compression, frictional state concept, dilatant failure state

## Abstract

The dilatant failure state in the stress ratio–plastic dilatancy relationship is crucial in the frictional state concept. This article presents a methodology for determining the dilatant failure state from the results of drained triaxial compression tests. For geomaterials undergoing dilative behavior during shearing, the dilatant failure state corresponds to the state of minimum plastic dilatancy. For contractive behavior, the proposed calculation procedure can be used to determine the dilatant failure state. In general, the dilatant failure state and the failure state are different. The points representing dilatant failure states in the stress ratio–plastic dilatancy plane can be approximated by a straight line (dilatant failure state line). Grain crushing and debonding during shearing significantly increase the slope of this line. The intersection of this line with the vertical axis determines the critical frictional state angle for granular materials. The dilatant failure state, with previously defined natural state parameter, allows for the determination of the critical state angle void ratio without physically reaching the critical frictional state. In general, given the fact that the dilatant failure state proceeds the failure state, the stresses and strains in the shearing specimen can be determined more accurately than in the ultimate, critical state. The frictional state concept may be viewed as an extension of the critical state concept developed over fifty years ago.

## 1. Introduction

Dilatancy is a phenomenon that occurs in all geomaterials, characterized by a change in volume during shear deformation. In addition to the type of geomaterial and its structure [1,2,3], dilatancy depends on the stress level, stress and strain history [4,5,6], shear deformation mode [7,8], water content [9,10,11], stress path [12,13], and grain shape [14]. The minimum value of dilatancy for sands and over-consolidated remolded clay represents the maximum stress ratio (failure) state [15,16,17]. However, for some geomaterials, such as artificially and naturally cemented soils, a lack of coexistence between the minimum dilatancy and failure states (FSs) has been observed [2,12,18].

Various relationships between the stress ratio and plastic dilatancy have been proposed (e.g., [19,20,21,22]). The stress ratio–plastic dilatancy relationship is a crucial component of classical elastoplastic models of geomaterials [19,21,23]. In these models, the relationship between the stress ratio and plastic dilatancy usually determines the plastic potential function [24]. The phase transformation state, defined as the state in which the soil behavior changes from contraction to dilation [25,26], is another characteristic state of soil that can be identified from the relationship between stress ratio and dilatancy.

Based on dissipated energy balance considerations, a linear stress ratio–plastic dilatancy relationship has previously been obtained [26]. That relationship is defined by the critical frictional state angle ($\phi^{o}$), stress path, and two (α, β) parameters of the frictional state concept (FSC). For drained triaxial compression, a stress ratio–plastic dilatancy relationship with constant parameter values *α* and *β* can be used to approximate the behavior of soil during different stages of shearing [26].

In this study, published drained triaxial compression test data for various geomaterials are analyzed to obtain the stress ratio–plastic dilatancy relationship at the current state of shearing. This relationship is represented as a curve in the $\eta -D^{p}$ plane. A methodology for calculating the slope of the tangent (A) and the curvature (ϰ) at any point on this curve is presented. The state corresponding to the local maximum curvature closest to the failure state (FS) is called the *dilatational failure state* (DFS). A straight line approximating the DFS is called the *dilatant failure state line* (DFSL). The intersection point of this line with the vertical axis of the $\eta -D^{p}$ plane determines the critical frictional angle of the analyzed geomaterials.

## 2. General Stress Ratio–Plastic Dilatancy Relationship

The general stress ratio–plastic dilatancy relationship has the form [26]

(1) $$\eta =Q-AD^{p},$$

where

(2) $$\eta =q/p^{\prime },$$

(3) $$Q=M^{o}-\alpha A^{o},$$

(4) $$A=\beta A^{o},$$

(5) $$A^{o}=1-M^{o}/\left(\delta q/\delta p\prime \right),$$

(6) $$D^{p}={\delta \varepsilon }_{\upsilon }^{p}/{\delta \varepsilon }_{q}^{p},$$

(7) $$M^{o}=M_{c}^{o}\;g\left(\theta^{o}\right),$$

(8) $$M_{c}^{o}=6\sin\phi^{o}/\left(3-\sin\phi^{o}\right),$$

(9) $$g\left(\theta^{o}\right)=\left(3\sin\phi^{o}\right)/2\left\{\sqrt{3\;}\cos\theta^{o}-\sin\phi^{o}\sin\theta^{o}\right\},$$

(10) $$q=\left|\sqrt{3\;J_{2}}\right|,$$

(11) $$p^{\prime }=\frac{1}{3}\sigma_{kk}^{\prime }=\frac{1}{3}\left(\sigma_{1}^{\prime }+\sigma_{2}^{\prime }+\sigma_{3}^{\prime }\right),$$

(12) $${\delta \varepsilon }_{\upsilon }^{p}=\delta \varepsilon_{kk}^{p}={\delta \varepsilon }_{1}^{p}+{\delta \varepsilon }_{2}^{p}+{\delta \varepsilon }_{3}^{p},$$

(13) $${\delta \varepsilon }_{q}^{p}=\left|\sqrt{\frac{4}{3}\;J_{\varepsilon_{2}}}\right|,$$

(14) $$J_{2}=\frac{1}{2}s_{ij}\;s_{ij},$$

(15) $$J_{\varepsilon_{2}}=\frac{1}{2}\delta e_{ij}^{p}\;\delta e_{ij}^{p},$$

(16) $$s_{ij}=\sigma_{ij}^{\prime }-p\prime \delta_{ij},$$

(17) $$\delta e_{ij}^{p}=\delta \varepsilon_{ij}^{p}-\frac{1}{3}\varepsilon_{\upsilon }^{p}\;\delta_{ij},$$

(18) $${\delta \varepsilon }_{\upsilon }^{p}={\delta \varepsilon }_{\upsilon }-{\delta \varepsilon }_{\upsilon }^{e},$$

(19) $${\delta \varepsilon }_{q}^{p}={\delta \varepsilon }_{q}-{\delta \varepsilon }_{q}^{e},$$

(20) $${\delta \varepsilon }_{\upsilon }^{e}=\delta p\prime /K,$$

(21) $${\delta \varepsilon }_{q}^{e}=\delta q/3G,$$

$\phi^{o}$ is the critical frictional state angle, θ is the Lode angle for stress, α and β are the FSC parameters, and K and G are the elastic bulk and shear modulus, respectively [26]. The elastic parameters are calculated using the following equations:

(22) $$G=G_{0}\frac{{\left(2.97-e\right)}^{2}}{\left(1+e\right)}{\left(\frac{p\prime }{p_{a}}\right)}^{0.5},$$

(23) $$K=\frac{2}{3}\;\frac{1+\nu }{1-2\nu }\;G,$$

or

(24) $$K=\frac{\left(1+e\right)p^{\prime }}{\kappa },$$

where $G_{0}$ is the parameter of the geomaterial, κ is the parameter of a Cam–Clay model, ν is Poisson’s ratio, e is the void ratio, and $p_{a}$ = 101 kPa is the atmospheric pressure.

For conventional drained triaxial compression, θ = $\theta^{o}$ = π/6, δq/δp′ = 3 [27], and:

(25) $$M^{o}=M_{c}^{o},$$

(26) $$A^{o}=A_{c}^{o}=1-\frac{1}{3}M_{c}^{o},$$

(27) $$q=\sigma_{1}^{\prime }-\sigma_{3}^{\prime }\;,$$

(28) $$p^{\prime }=\frac{1}{3}\;\left(\sigma_{1}^{\prime }+2\sigma_{3}^{\prime }\right),$$

(29) $$\varepsilon_{\upsilon }^{p}=\varepsilon_{1}^{p}+2\varepsilon_{3}^{p},$$

(30) $$\varepsilon_{q}^{p}=\frac{2}{3}\left(\varepsilon_{1}^{p}-\varepsilon_{3}^{p}\right).$$

The same relationships are assumed for drained triaxial compression with p′=constant stress path.

**Figure 1:**
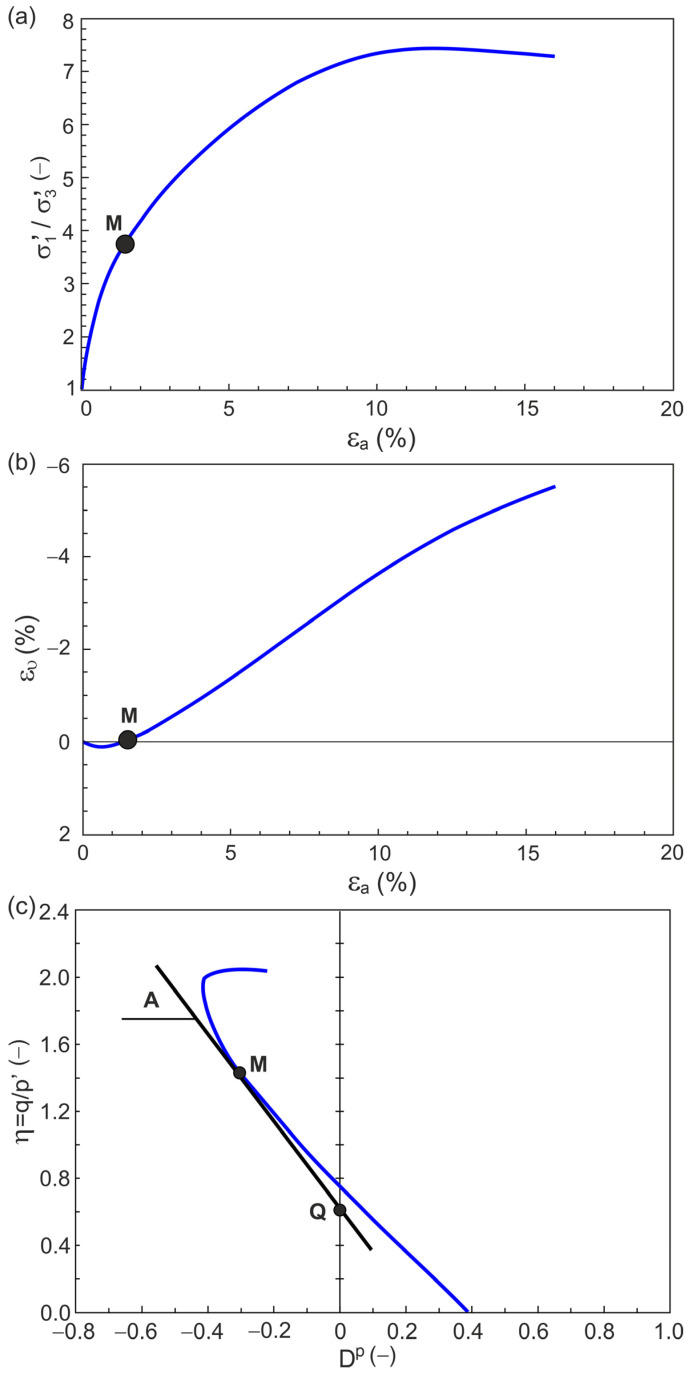
Examples of typical relationships for a geomaterial under shear in the planes: (**a**) $\sigma_{1}^{\prime }/\sigma_{3}^{\prime }-\varepsilon_{a}$; (**b**) $\varepsilon_{\upsilon }-\varepsilon_{a}$; (**c**) $\eta -D^{p}$.

Figure 1 shows the relationships $\sigma_{1}^{\prime }/\sigma_{3}^{\prime }-\varepsilon_{a}$ and $\varepsilon_{\upsilon }-\varepsilon_{a}$ for a conventional drained triaxial compression test conducted by Michalowski and Čermák [28] on sand with 2% fibers at $\sigma_{3}^{\prime }\;=$ 100 kPa.

The $\eta -D^{p}$ relationship for this test is shown in Figure 1. A tangent line can be calculated at any non-singular point *M* (Figure 1) of the $\eta -D^{p}$ relationship curve. The slope of this tangent line to the horizontal axis is [29]:

(31) $$A=-\frac{\delta \eta }{\delta D^{p}}.$$

The tangent line intersects the vertical axis at the point Q (Figure 1c). Hence, the parameters Q (Equation (3)) and A (Equation (4)) of the stress ratio–plastic dilatancy relationship (Equation (1)) have a simple geometric interpretation.

## 3. Curvature of the Stress Ratio–Plastic Dilatancy Relationship Curve

**Figure 2:**
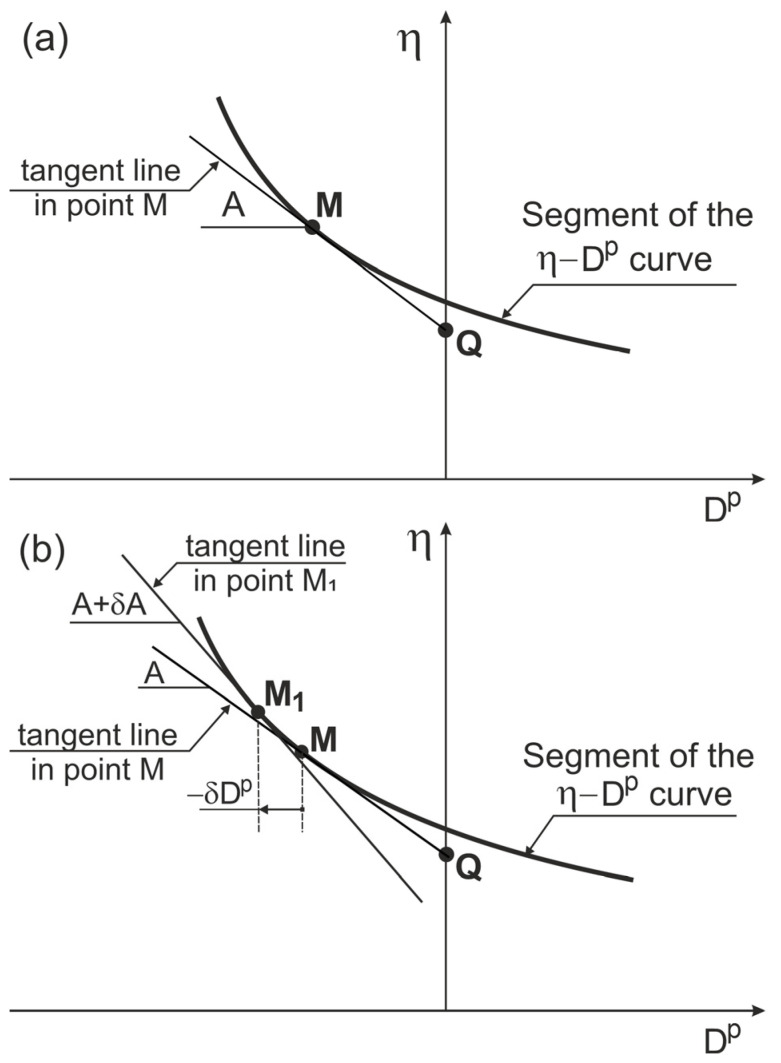
Slope and curvature of the stress ratio–plastic dilatancy relationship curve: (**a**) slope; (**b**) curvature.

At any non-singular point (*M*) of the stress ratio–plastic dilatancy relationship curve (Figure 2), an absolute curvature can be calculated as follows [29]:

(32) $$\varkappa =\left|\frac{\delta A}{\delta D^{p}}\right|.$$

To calculate the slope (A) and curvature (ϰ), it is convenient to segmentally approximate the stress ratio (η) and plastic dilatancy ($D^{p}$) using analytical functions (e.g., polynomials) of $\varepsilon_{q}^{p}$, as follows:

(33) $$\eta =f_{\eta }\;\left(\varepsilon_{q}^{p}\right),$$

(34) $$D^{p}=f_{D}\;\left(\varepsilon_{q}^{p}\right).$$

Then

(35) $$A=-\frac{\delta \eta }{\delta D^{p}}=-\frac{\left\{\delta f_{\eta }\;\left(\varepsilon_{q}^{p}\right)\right\}\;/\;\delta \varepsilon_{q}^{p}}{\left\{\delta f_{D}\;\left(\varepsilon_{q}^{p}\right)\right\}\;/\;\delta \varepsilon_{q}^{p}},$$

and

(36) $$\varkappa =\left|\frac{\delta A}{\delta D^{p}}\right|=\left|\frac{\frac{\delta^{2}f_{\eta }\left(\varepsilon_{q}^{p}\right)}{\delta \varepsilon_{q}^{p2}}\;\frac{\delta f_{D}\left(\varepsilon_{q}^{p}\right)}{{\delta \varepsilon }_{q}^{p}}-\frac{\delta f_{\eta }\left(\varepsilon_{q}^{p}\right)}{{\delta \varepsilon }_{q}^{p}}\;\frac{\delta^{2}f_{D}\left(\varepsilon_{q}^{p}\right)}{\delta \varepsilon_{q}^{p2}}}{\;{\left\{\left[\delta f_{D}\left(\varepsilon_{q}^{p}\right)\right]\;/\;\delta \varepsilon_{q}^{p}\right\}}^{2}\;}\right|.$$

The state of shear deformation in which the increment of plastic dilatancy changes from positive to negative is called the *plastic dilatancy increment transitional state* (PDITS). In the PDITS, we have:

(37) $$\frac{\delta D^{p}}{{\delta \varepsilon }_{q}^{p}}=\frac{\delta f_{D}\left(\varepsilon_{q}^{p}\right)}{{\delta \varepsilon }_{q}^{p}}=0.$$

The slope of the tangent line and the curvature of the stress ratio–plastic dilatancy relationship curve cannot be calculated using Equations (35) and (36), respectively. The PDITS corresponds to the minimum plastic dilatancy state for the dilative behavior of the geomaterial during shear. The denominator of Equations (35) and (36) is also equal to zero in the state of shear deformation with constant plastic dilatancy, $D^{p}=a=\;$constant ($\varepsilon_{\upsilon }^{p}=a\cdot \varepsilon_{q}^{p}$). This state is called the *constant plastic dilatancy state* (CPDS). In the near pre- and post-PDITS states, as well as at the onset of the CPDS state, the values of ${\delta D}^{p}/\delta \varepsilon_{q}^{p}$ are minimal, while the A and (ϰ) values are very large.

In this paper, we do not consider cases involving shear deformation with constant stress ratio and zero plastic dilatancy, where the nominator and denominator of Equations (35) and (36) have zero values and the slope and curvature of the $\eta -D^{p}$ relationship curve are not defined.

The asymptotic states, the critical state for loose granular materials and normally consolidated clays, and the steady states are the failure states for which the nominator and denominator of Equations (35) and (36) are equal to zero, may but don’t have to be dilatant failure states.

## 4. Methodology

Selected results of drained triaxial compression tests of some geomaterials presented in the literature are digitized and analyzed. Based on these digitized values of stresses and volumetric strains as a function of strain, the relationships $\eta -\varepsilon_{q}^{p}$, $\varepsilon_{\upsilon }-\varepsilon_{q}^{p}$ and $D^{p}-\varepsilon_{q}^{p}$ were calculated.

The obtained $\eta -\varepsilon_{q}^{p}$ and $D^{p}-\varepsilon_{q}^{p}$ relationships were approximated segmentally using polynomials. Based on the authors’ experience, more accurate locations of the points representing the dilatant failure state in the $\eta -\varepsilon_{q}$, $\varepsilon_{\upsilon }-\varepsilon_{q}$, $\eta -D^{p}$, and $D^{p}-\varepsilon_{q}^{p}$ planes were obtained without directly analyzing the $\eta -D^{p}$ relationship, but by analyzing the $\eta -\varepsilon_{q}^{p}$ and $D^{p}-\varepsilon_{q}^{p}$ relationships using Equations (35) and (36). To avoid overfitting, the experimental $\eta -\varepsilon_{q}^{p}$ and $D^{p}-\varepsilon_{q}^{p}$ relationships were approximated segmentally using polynomials of degree up to 5 or 6. If the approximation accuracy was insufficient, the number of segments was increased. In each segment, the approximation accuracy was determined in a conventional manner. Special attention was paid to the continuity in the η and $D^{p}$ values, as well as their increments δη and $\delta D^{p}$ at the contact points between adjacent segments. The values of the tangent line slope (A), and curvature (ϰ) of the curve of stress ratio–plastic dilatancy at any non-singular points were then calculated.

**Figure 3:**
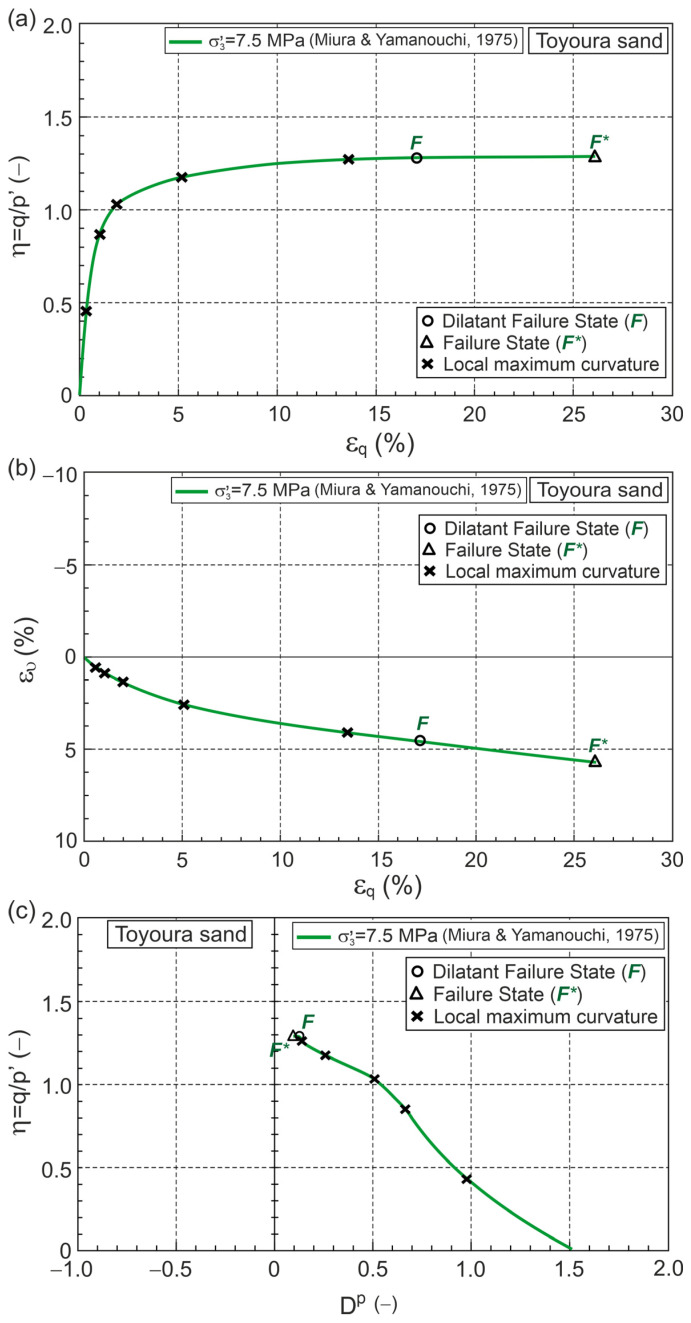

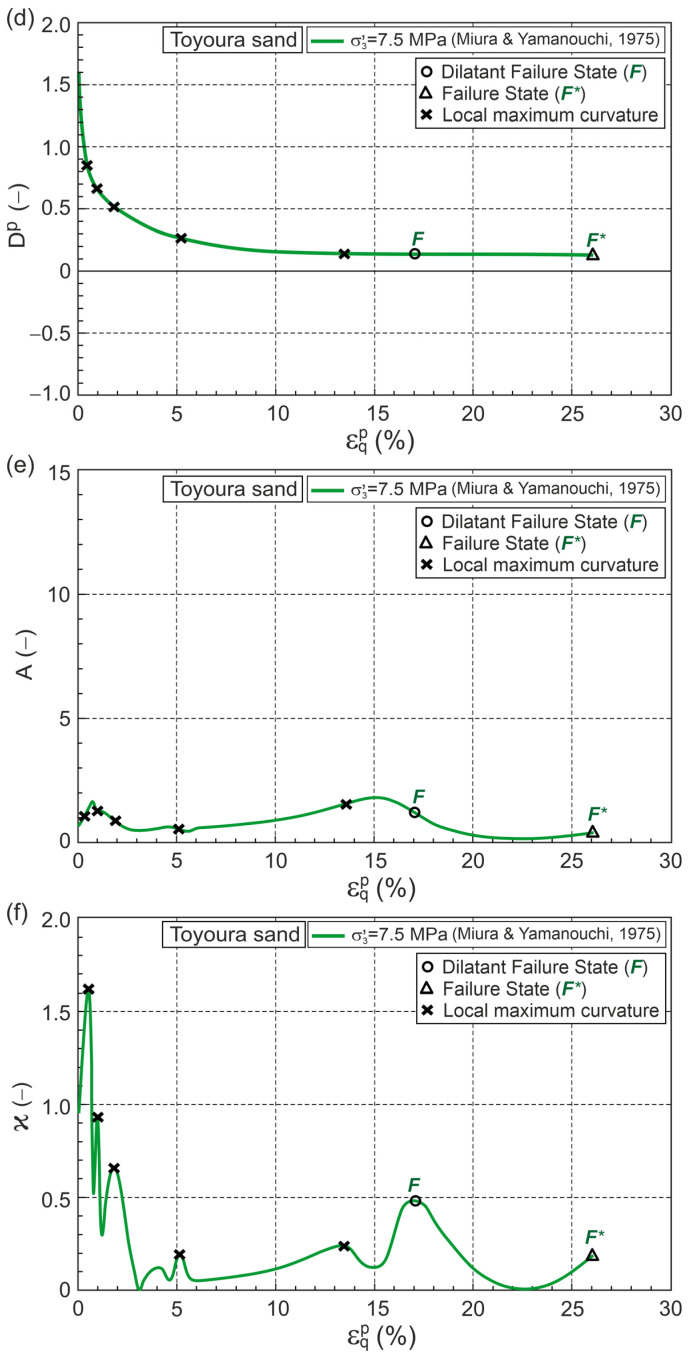
Relationship for Toyoura sand during drained triaxial compression under high confining pressure (Test data from [30]) in planes: (**a**) $\eta -\varepsilon_{q}$; (**b**) $\varepsilon_{\upsilon }-\varepsilon_{q}$; (**c**) $\eta -D^{p}$; (**d**) $D^{p}-\varepsilon_{q}^{p}$; (**e**) $A-\varepsilon_{q}^{p}$; (**f**) $\varkappa -\varepsilon_{q}^{p}$.

As an example, the obtained relationship in the planes $\eta -\varepsilon_{q}$, $\varepsilon_{\upsilon }-\varepsilon_{q}$, $D^{p}-\varepsilon_{q}^{p}$, $A-\varepsilon_{q}^{p}$, and $\varkappa -\varepsilon_{q}^{p}$ for a conventional drained triaxial compression test on Toyoura sand, performed by Miura and Yamanouchi [30], as shown in Figure 3.

The maximum local curvatures of the $\eta -D^{p}$ relationship were determined and marked on the $\varkappa -\varepsilon_{q}^{p}$ curve (Figure 3f). The points corresponding to the local maximum curvature and failure state are shown in Figure 3.

## 5. Dilatant Failure State

The DFS is the state in which the local maximum curvature is closest to the failure state (FS). The DFS and FS are marked in Figure 3 as points F and F*, respectively. The DFS closest to FS does not always mean close, especially on the $\eta -\varepsilon_{q}$, $\varepsilon_{\upsilon }-\varepsilon_{q}$, $D^{p}-\varepsilon_{q}^{p}$, $A-\varepsilon_{q}^{p}$, and $\varkappa -\varepsilon_{q}^{p}$ planes (Figure 3).

In this paper, the results of selected drained triaxial compression tests were analyzed, and the DFSs were determined. The DFSL is described by the general stress ratio–plastic dilatancy relationship in Equation (1) as

(38) $$\eta_{F}=Q_{F}-A_{F}D_{F}^{p},$$

where

(39) $$Q_{F}=M_{c}^{o}+\alpha_{F}{\;A}_{c}^{o},$$

(40) $$A_{F}=\beta_{F}A_{c}^{o},$$

where $\eta_{F}$, $D_{F}^{p}$ are the stress ratio and plastic dilatancy at the DFS, respectively. The parameters $\alpha_{F}$ and $\beta_{F}$ are FSC parameters describing the DFSL. Equation (26) defines $A_{c}^{o}$ independently of the stress path.

In each analysis, it was assumed that $\alpha_{F}=\;$0. Hence, $Q_{F}=M_{c}^{o}$, and the critical frictional state angle ($\phi^{o}$) can be calculated from the equation

(41) $$\sin\phi^{o}=\frac{3M_{c}^{o}}{6+M_{c}^{o}}.$$

In the FSC [27], $\phi^{o}$ is independent of deformation mode (Lode angle). To finally prove the correctness of the obtained $\phi^{o}$ values, triaxial extension tests should be performed, and the resulting data should be analyzed in a similar way.

## 6. Dilatant Failure States in Drained Triaxial Compression Tests of Some Geomaterials

### 6.1. Toyoura Sand

**Figure 4:**
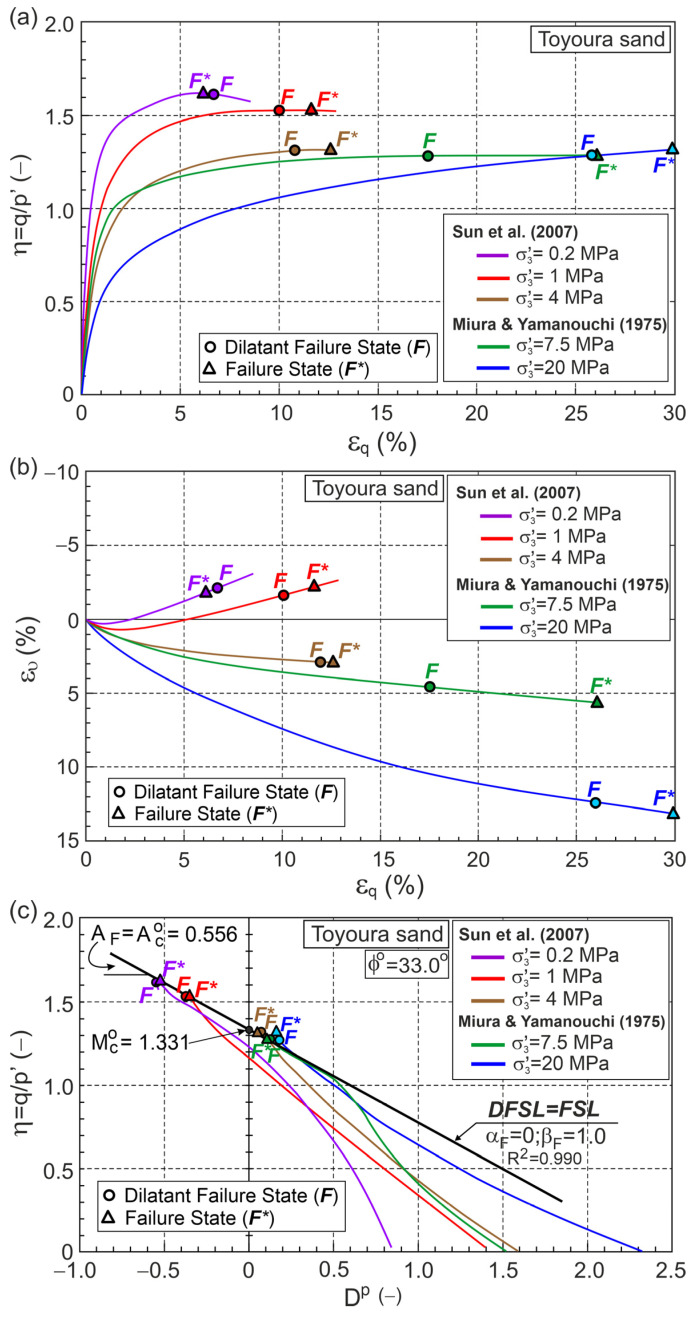

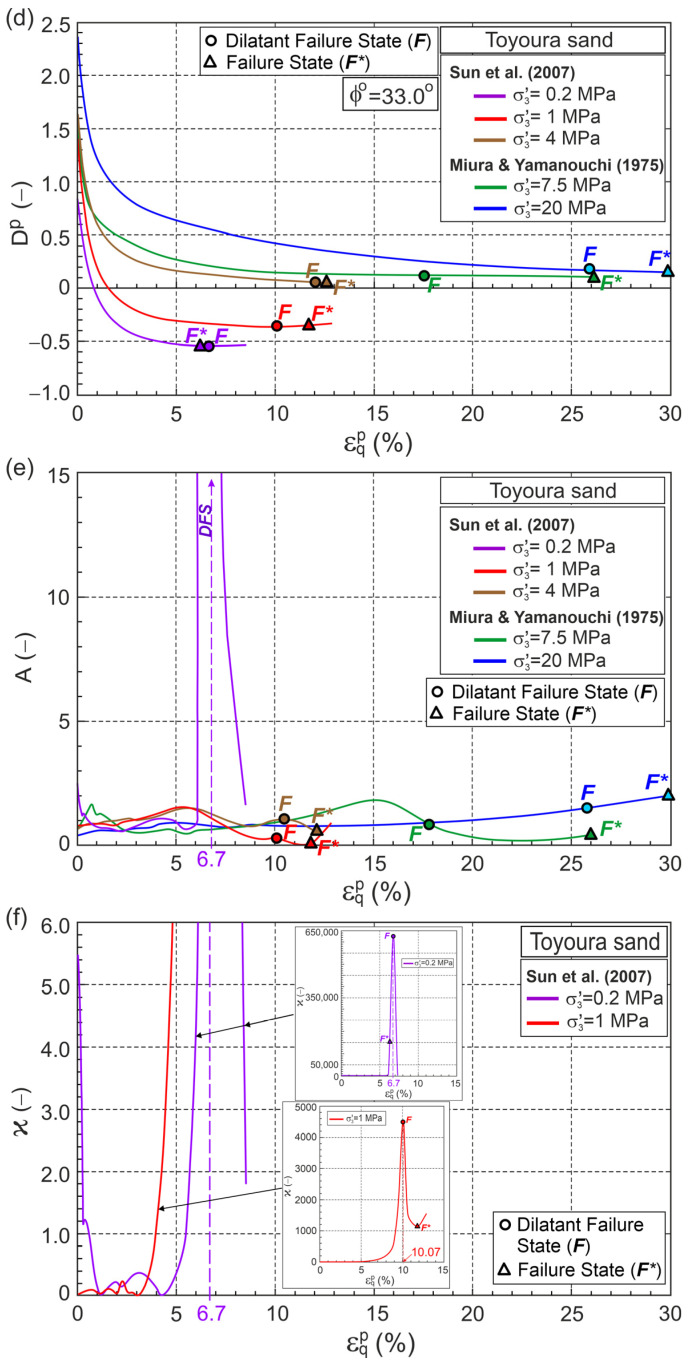

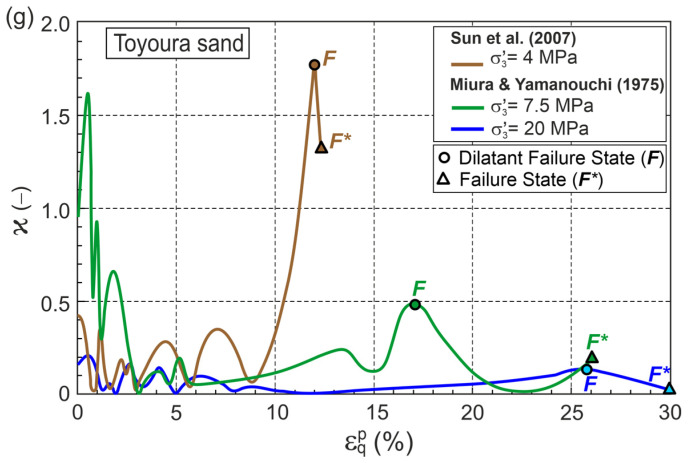
Relationships for Toyoura sand during drained triaxial compression (Test data from [30,31]) in planes: (**a**) $\eta -\varepsilon_{q}$; (**b**) $\varepsilon_{\upsilon }-\varepsilon_{q}$; (**c**) $\eta -D^{p}$; (**d**) $D^{p}-\varepsilon_{q}^{p}$; (**e**) $A-\varepsilon_{q}^{p}$; (**f**) $\varkappa -\varepsilon_{q}^{p}$ for $\sigma_{3}^{\prime }=0.2$ MPa and 1 MPa; (**g**) $\varkappa -\varepsilon_{q}^{p}$ for $\sigma_{3}^{\prime }=4$, 7.5, and 20 MPa.

Fine-grained quartz Toyoura sand has been extensively tested in geotechnical laboratories. Data from five conventional drained triaxial compression tests selected from the literature were analyzed in this work, comprising. Three tests at low and medium stress levels, conducted by Sun et al. [31], and two tests at high stress levels, conducted by Miura and Yamanouchi [30]. According to the methodology presented above, with the initial and elastic parameters shown in Table A1, the relationships $\eta -\varepsilon_{q}$, $\varepsilon_{\upsilon }-\varepsilon_{q}$, $D^{p}-\varepsilon_{q}^{p}$, $A-\varepsilon_{q}^{p}$, and $\varkappa -\varepsilon_{q}^{p}$ were obtained as shown in Figure 4.

Points F and F* represent the DFS and FS, respectively, and are marked on the graph. DFSs slightly precede FSs for the dilative behavior during shear, whereas for tests performed at medium and high stress levels, which exhibit contractive behavior, DFSs significantly precede FSs (Figure 4).

**Figure 5:**
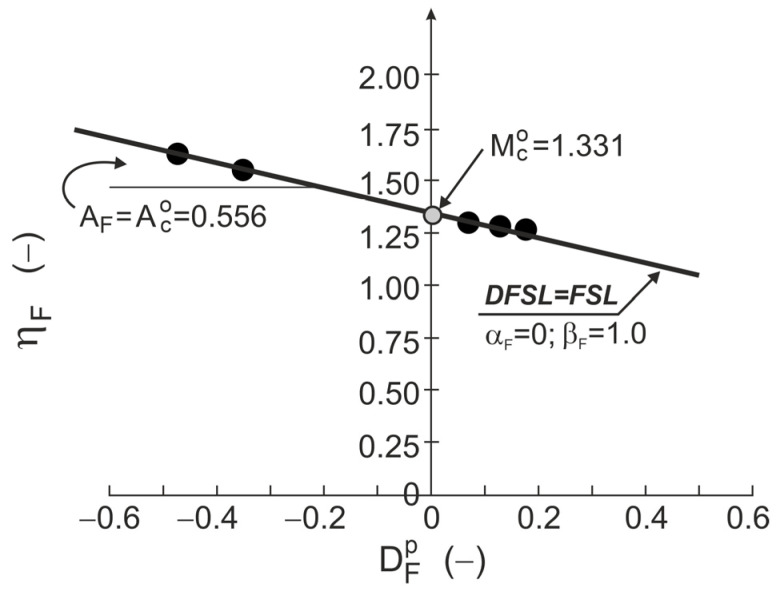
FSL and DFSL for drained triaxial compression of Toyoura sand.

The DFSL closely approximates points representing DFSs on the $\eta -D^{p}$ plane (Figure 4c and Figure 5). This DFSL intersects the vertical axis at the point $\eta =Q_{F}=M_{c}^{o}=$1.331 ($\phi^{o}=33{}^{\circ}$, $\alpha_{F}=\;$0) with slope $A_{F}=A_{c}^{o}=0.556$ ($\beta_{F}=$1.0). This means that the possible presence of grain crushing during drained triaxial compression shearing at high stress levels does not affect the slope and location of the DFS and $\phi^{o}=\phi_{cs}$ [30,31].

### 6.2. Crushed Concrete

**Figure 6:**
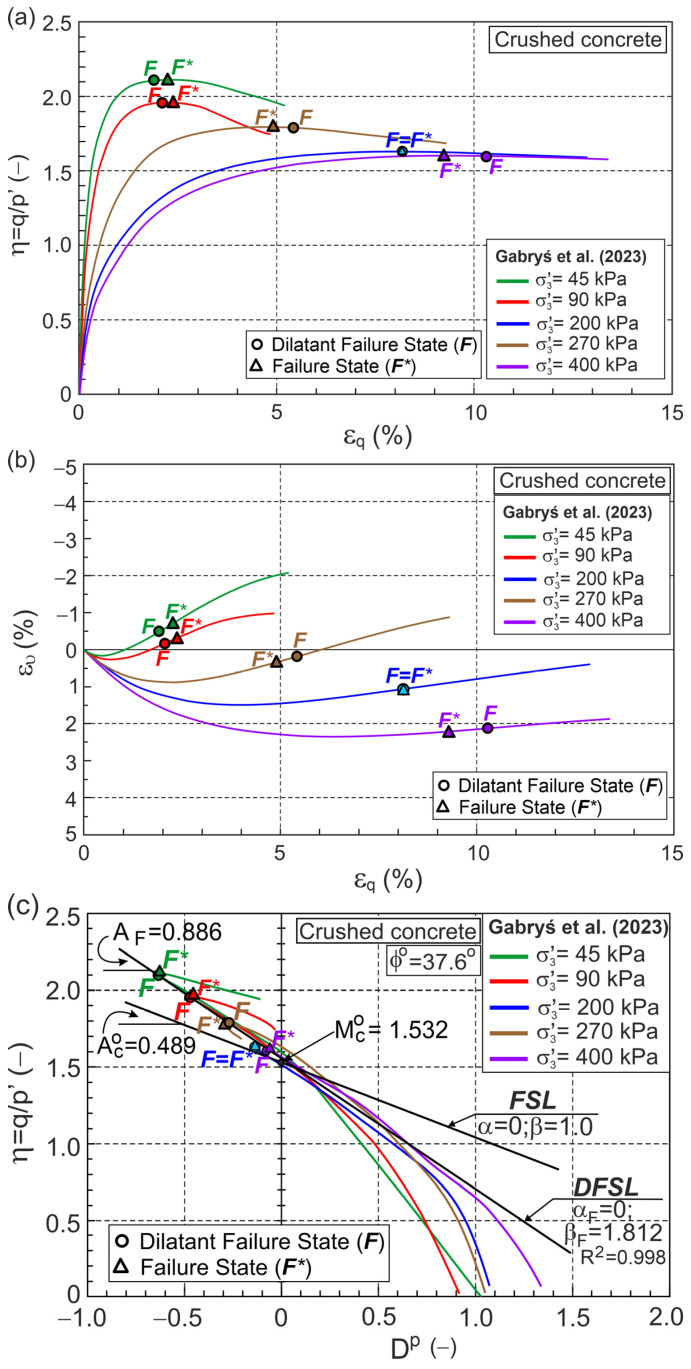

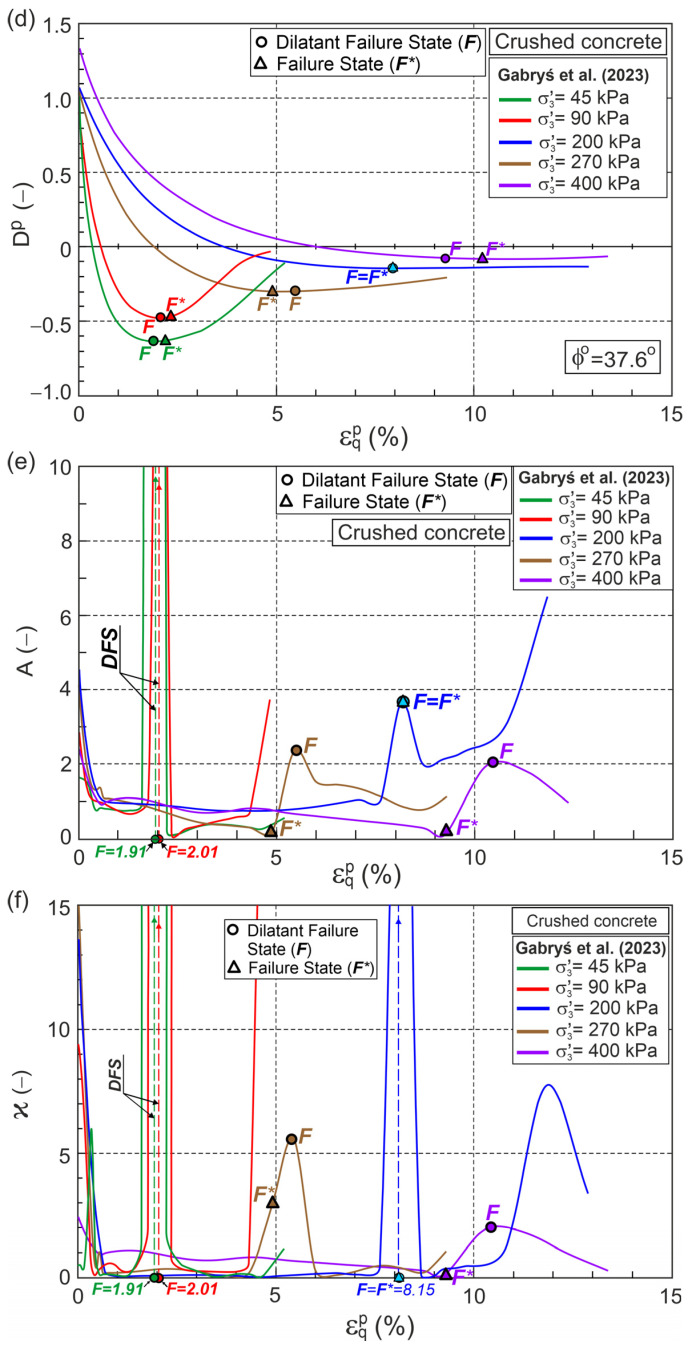
Relationships for crushed concrete during drained triaxial compression (Test data from [32]) in planes: (**a**) $\eta -\varepsilon_{q}$; (**b**) $\varepsilon_{\upsilon }-\varepsilon_{q}$; (**c**) $\eta -D^{p}$; (**d**) $D^{p}-\varepsilon_{q}^{p}$; (**e**) $A-\varepsilon_{q}^{p}$; (**f**) $\varkappa -\varepsilon_{q}^{p}$.

As a popular anthropogenic soil, crushed concrete was conventionally triaxially compressed under drained conditions [32]. Grains of crushed concrete are very sharp-edged. As a result, high stresses occur at the grain contact points, and the material is susceptible to crushing even at low stress levels. Data from five tests were analyzed using the elastic and initial state parameters shown in Table A1. The results of the analysis are shown in Figure 6.

Dilative behavior was observed in all the tests, and the maximum values of A and ϰ correspond to the minimum plastic dilatancy states. For $\sigma_{3}^{\prime }=$ 45 and 90 kPa, DFS precedes FS, whereas for $\sigma_{3}^{\prime }=$ 200 and 400 kPa, FS precedes DFS, and for $\sigma_{3}^{\prime }=$ 270 kPa, DFS and FS are the same states. The DFSL, the best fitting DFS in the $\eta -D^{p}$ plane, intersects the vertical axis at $\eta =Q_{F}=$1.532 with slope is $A_{F}=$0.886 (Figure 6c). Assuming $\alpha_{F}$ = 0 ($Q_{F}=M_{c}^{o}$), we have $\phi^{o}$ = 37.6° and $\beta_{F}=$1.812. $\beta_{F}$ > 1, or more precisely ($\beta_{F}-1$) > 0, quantifies the influence of grain crushing dissipation energy on the relationship $\eta -D^{p}$ [26].

### 6.3. Dog’s Bay Sand

**Figure 7:**
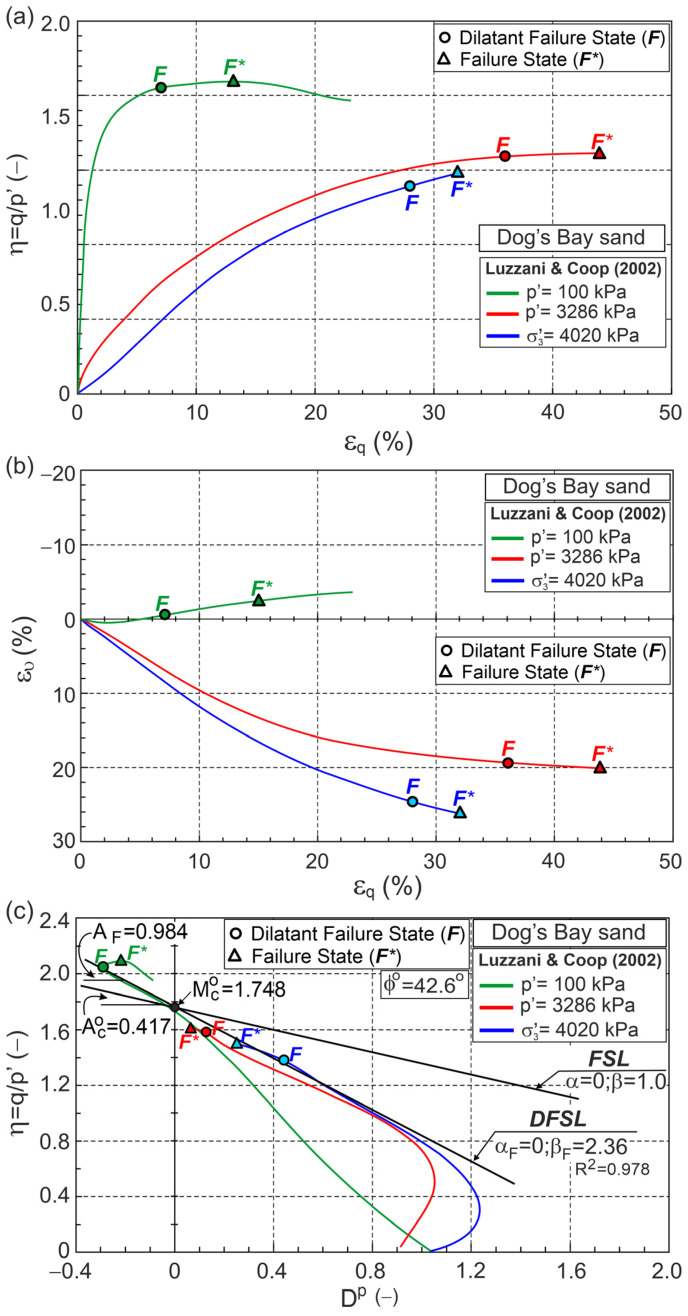

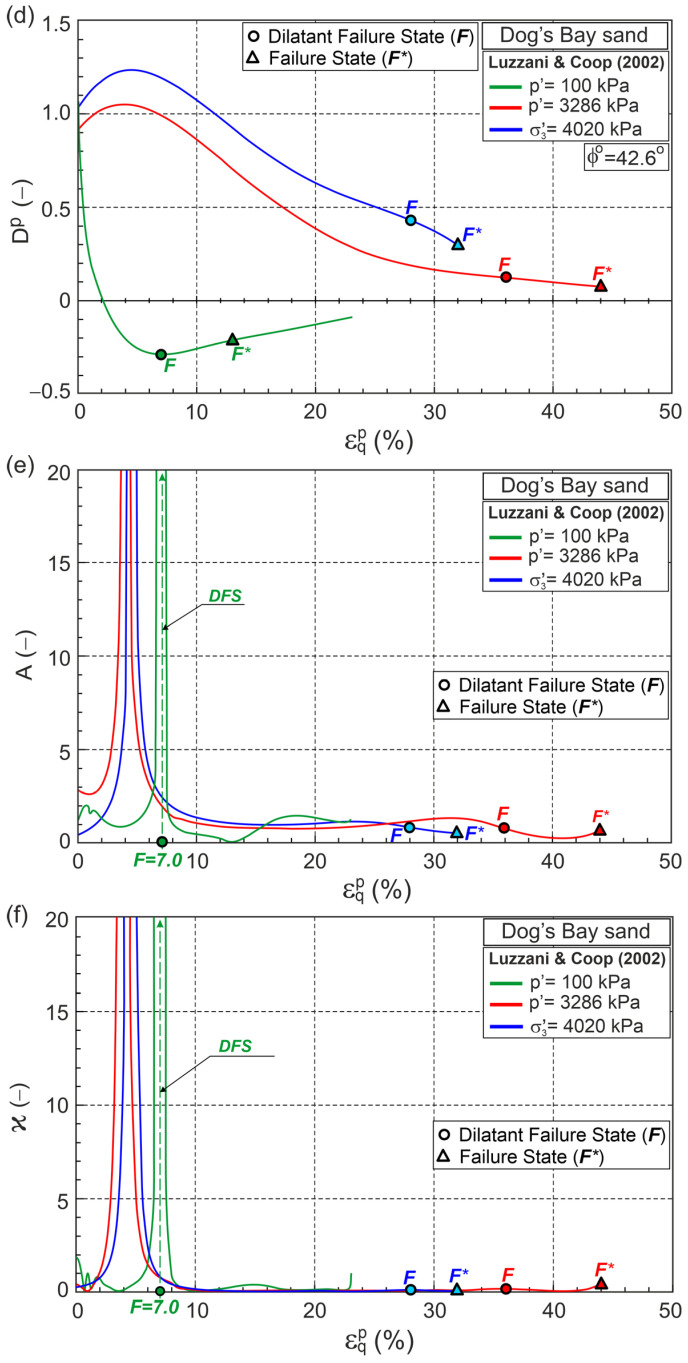
Relationships for Dog’s Bay sand during drained triaxial compression (Test data from [34]) in planes: (**a**) $\eta -\varepsilon_{q}$; (**b**) $\varepsilon_{\upsilon }-\varepsilon_{q}$; (**c**) $\eta -D^{p}$; (**d**) $D^{p}-\varepsilon_{q}^{p}$; (**e**) $A-\varepsilon_{q}^{p}$; (**f**) $\varkappa -\varepsilon_{q}^{p}$.

Dog’s Bay sand is a biogenic carbonate sand, essentially consisting of foraminifera and Mollusca shells [33]. Dog’s Bay sand grains are easily crushed. Coop [33] extensively tested this sand. Drained and undrained triaxial compression tests on isotropically and $K_{0}-$consolidated samples were performed. In the present work, three test results were selected for analysis: two drained triaxial compression tests conducted at constant mean effective stresses of $p^{\prime }=$ 100 and 3286 kPa, and one conventional drained triaxial compression test performed at a constant $\sigma_{3}^{\prime }=\;$4020 kPa. The elastic and initial state parameters are shown in Table A1. The analysis results are presented in Figure 7.

The test at a constant mean stress p’= 100 kPa exhibits dilative behavior, while the other two tests at high stress levels exhibit contractive behavior. In all of the tests, DFS significantly precedes FS (Figure 7). DFSL intersects the vertical axis at $\eta =\;Q_{F}=$ 1.748 with slope $A_{F}=$ 0.984 (Figure 7c). Assuming that $\alpha_{F}=$ 0, we have $Q_{F}=M_{c}^{o}=$ 1.748. The critical frictional state angle $\phi^{o}=\;$42.6° is greater than $\phi_{cs}=\;$40.3° [33,34]. The FSC parameter $\beta_{F}=\;$2.36 (Figure 7c). DFSL closely approximates the DFSs for tests at different stress levels and stress paths. In the case of dilative behavior, DFS corresponds to a state with minimum plastic dilatancy.

### 6.4. Cemented Portaway Sand

**Figure 8:**
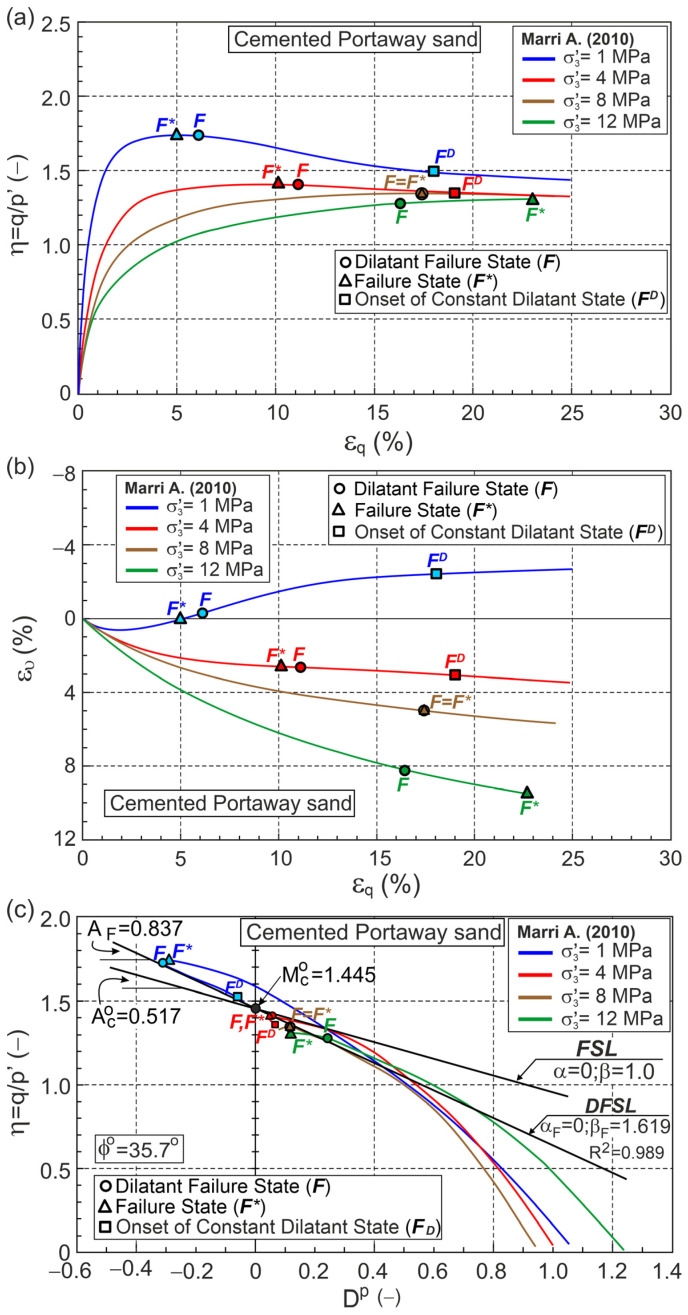

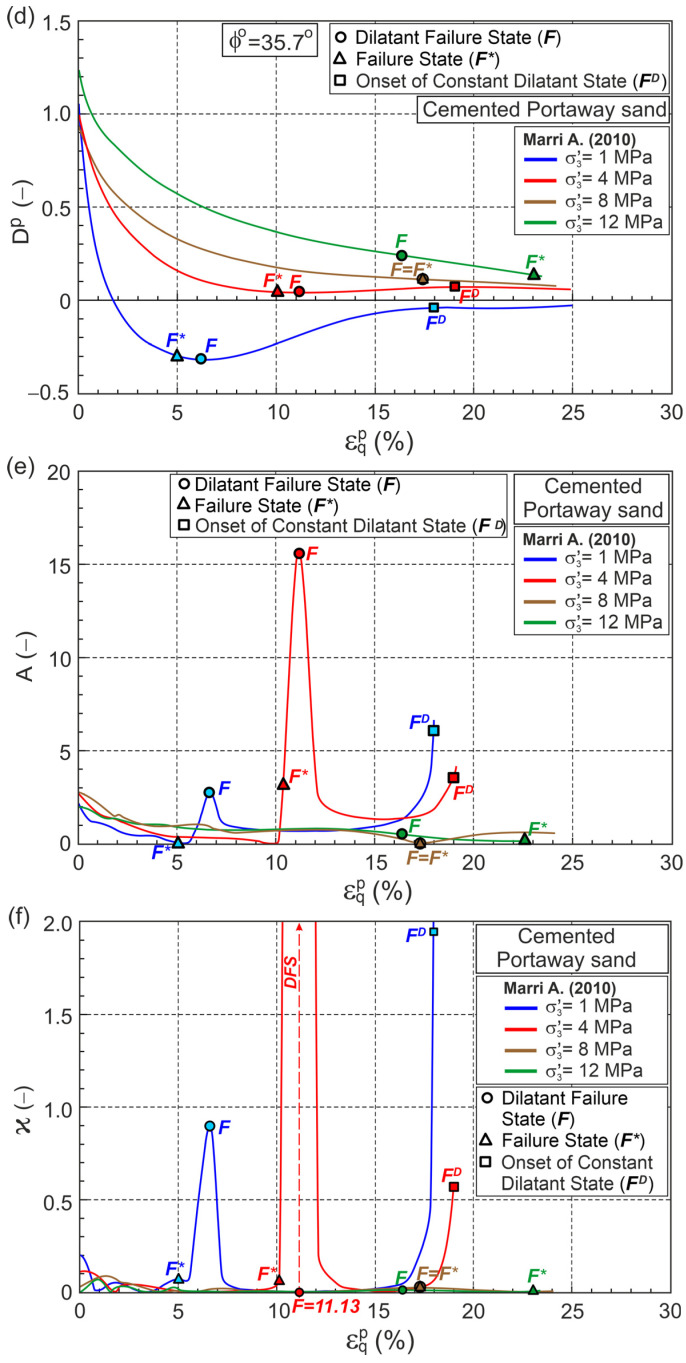
Relationships for cemented Portaway sand during drained triaxial compression (Test data from [35]) in planes: (**a**) $\eta -\varepsilon_{q}$; (**b**) $\varepsilon_{\upsilon }-\varepsilon_{q}$; (**c**) $\eta -D^{p}$; (**d**) $D^{p}-\varepsilon_{q}^{p}$; (**e**) $A-\varepsilon_{q}^{p}$; (**f**) $\varkappa -\varepsilon_{q}^{p}$.

Marri [35] tested Portaway sand with different values of the cement content. Four conventional drained triaxial compression test results with 5% of cement content were selected for analysis here. The calculation results are shown in Figure 8 and Table A1.

Dilative behavior is observed at a confining pressure of $\sigma_{c}=\;$1 MPa. Three tests performed at confining pressures of $\sigma_{c}=\;$4, 8, and 12 MPa exhibit contractive behavior, and FS precedes DFS for tests at confining pressures of $\sigma_{c}=\;$1 and 4 MPa (Figure 8). In these tests, DFSs were observed at significant shear strains. The DFS and FS are equivalent for the test at a confining pressure of $\sigma_{c}=\;$8 MPa, whereas for the test at a confining pressure of $\sigma_{c}=\;$12 MPa, DFS significantly precedes the FS (Figure 8). The points marked $F^{D}$ represent the onset of a constant dilatant state (CDS) for which the slope (A) and curvature (ϰ) of the $\eta -D^{p}$ curve cannot be calculated. DFSL closely approximates the DFSs, intersecting the vertical axis at $\eta =Q_{F}=\;$1.449 with slope $A_{F}=\;$0.837 (Figure 8c). Assuming $\alpha_{F}=\;$0, $M_{c}^{o}=\;$1.449 corresponds to the critical frictional state angle $\phi^{o}=\;$35.7°, the slope of the FSL $A_{c}^{o}=\;$0.517 and $\beta_{F}=\;$1.619. The value ($\beta_{F}-1$) = 0.619 > 0 represents the influence of debonding on the relationship for DFSs for Portaway cemented sand with 5% cement. The correctness of the parameters obtained for the FSC ($\phi^{o}$, $\alpha_{F}$, $\beta_{F}$) of cemented sand should be verified by triaxial extension tests and an analog analysis of the results.

## 7. Conclusions

(1) The methodology presented here for determining DFS, based on the results of a drained triaxial compression test, is effective but time-consuming.
(2) For dilative behavior, the DFS corresponds to the state of minimum plastic dilatancy. For contractive behavior, the DFS must be determined using the calculation procedure introduced in this work.
(3) The distance between DFS and FS is not always small, especially in the case of contractive behavior.
(4) The DFS is essential for describing the behavior of geomaterials during shear.
(5) A value ($\beta_{F}$ − 1) > 0 represents the effect of grain crushing or debonding at the DFS.
(6) DFSL and FSL are equivalent when there are no crushing and debonding effects.
(7) According to the FSC, the critical frictional state angle is independent of the deformation mode. Hence, the FSC parameters obtained from triaxial compression tests should be verified through triaxial extension tests, especially for bonded geomaterials.

## Data Availability

The original contributions presented in this study are included in the article. You can contact the corresponding authors if you have any other questions.

## Appendix A

**Table A1:** Parameters for the dilatant failure and failure states.

Geo-Material	Reference	Initial Conditions			DFS						FS			Elastic Parameters		
		$e_{i}$	$\sigma_{3}^{\prime }$	$p^{\prime }$	$M_{c}^{o}$	$\alpha_{F}$	$\beta_{F}$	$\eta_{F}$	$D_{F}^{p}$	$\varepsilon_{qF}^{p}$	$\eta_{F*}$	$D_{F*}^{p}$	$\varepsilon_{qF*}^{p}$	$G_{0}$	κ	ν
		-	kPa	kPa	-	-	-	-	-	%	-	-	%	MPa	-	-
Toyoura sand	Sun et al. (2007) [31]	0.680	200	-	1.331	0	1.000	1.614	−0.543	6.70	1.620	−0.541	6.10	25.0	-	0.3
		0.665	1000	-				1.526	−0.361	10.04	1.527	−0.348	11.7			
		0.644	4000	-				1.317	0.058	12.00	1.317	0.052	12.6			
	Miura & Yamanouchi (1975) [30]	0.564	7500	-				1.281	0.124	17.10	1.285	0.108	26.1			
		0.526	20,000	-				1.284	0.170	25.90	1.315	0.152	30.0			
Crushed concrete	Gabryś et al. (2023) [32]	0.572	45	-	1.532	0	1.812	2.106	−0.633	1.91	2.106	−0.628	2.23	26.5	-	0.15
		0.570	90	-				1.957	−0.478	2.06	1.958	−0.466	2.35			
		0.491	200	-				1.629	−0.145	8.15	1.629	−0.145	8.15			
		0.564	270	-				1.790	−0.300	5.44	1.794	−0.308	4.89			
		0.513	400	-				1.598	−0.083	10.29	1.599	−0.077	9.26			
Dog’s Bay sand	Luzzani & Coop (2002) [34]	1.584	-	100	1.748	0	2.36	2.054	−0.289	7.01	2.093	−0.213	13.0	-	0.0075	0.3
		1.075	-	3286				1.586	0.120	36.0	1.607	0.070	44.0			
		0.991	4020	-				1.388	0.429	28.0	1.477	0.292	32.0			
Cemented Portaway sand	Marri (2010) [35]	0.500	1000	-	1.449	0	1.619	1.733	−0.317	6.60	1.739	−0.297	4.99	40.0	-	0.35
		0.480	4000	-				1.403	0.041	11.12	1.406	0.043	10.13			
		0.470	8000	-				1.346	0.114	17.34	1.346	0.114	17.34			
		0.450	12,000	-				1.279	0.243	16.28	1.307	0.140	22.83			
